# The Antitumor Activity of TCR-Mimic Antibody-Drug Conjugates (TCRm-ADCs) Targeting the Intracellular Wilms Tumor 1 (WT1) Oncoprotein

**DOI:** 10.3390/ijms20163912

**Published:** 2019-08-12

**Authors:** Ying Shen, Yi-Ming Li, Jing-Jing Zhou, Zhan Zhou, Ying-Chun Xu, Wen-Bin Zhao, Shu-Qing Chen

**Affiliations:** Laboratory of Precision Medicine and Biopharmaceutics & Zhejiang Province Key Laboratory of Anti-Cancer Drug Research, College of Pharmaceutical Sciences, Zhejiang University, Hangzhou 310058, China

**Keywords:** TCR-mimic antibody, antibody-drug conjugates, Wilms tumor 1, human leukocyte antigen class I molecule, bispecific TCR-mimic antibody

## Abstract

Wilms tumor 1 (WT1) oncoprotein is an intracellular oncogenic transcription factor which is barely expressed in normal adult tissues but over expressed in a variety of leukemias and solid cancers. WT1-derived HLA-A*02:01 T cell epitope, RMFPNAPYL (RMF), is a validated target for T cell-based immunotherapy. We generated two T cell receptor mimic antibody-drug conjugates (TCRm-ADCs), ESK-MMAE, and Q2L-MMAE, against WT1 RMF/HLA-A*02:01 complex with distinct affinities, which mediate specific antitumor activity. Although ESK-MMAE showed higher tumor growth inhibition ratio in vivo, the efficacy of them was not so promising, which might be due to low expression of peptide/HLA targets. Therefore, we explored a bispecific TCRm-ADC that exerted more potent tumor cytotoxicity compared with TCRm-ADCs. Hence, our findings validate the feasibility of the presenting intracellular peptides as the targets of ADCs, which broadens the antigen selection range of antibody-based drugs and provides new strategies for precision medicine in tumor therapy.

## 1. Introduction

According to the Global Cancer Report of 2018, the latest global cancer statistics, there are 18.1 million new cancer cases and 9.6 million deaths worldwide. Cancer has become a global issue which severely threatens human health. In recent years, antibody-drug conjugates (ADCs) are considered as a promising agent for cancer therapy, which link potent cytotoxic agents to monoclonal antibodies (mAbs) through chemical linkages. ADCs specifically deliver cytotoxic agents into the tumor cells to exert antitumor effects and greatly reduce the side effects caused by the cytotoxic agents. Currently, there are five ADCs approved by the FDA (U.S. Food and Drug Administration) or EMA ( European Medicines Agency): Kadcyla^®^ (Genentech), Adcetris^®^ (Seattle Genetics), Mylotarg^®^ (Pfizer), Besponsa^®^ (Pfizer), and Polivy^®^ (Genentech), and more than 50 ADCs are in clinical trials [1]. Nevertheless, almost all traditional ADCs, including above five marketed ADCs, target extracellular or cell surface proteins which account for only a tiny fraction of the total cellular proteins [2,3]. Due to the existence of cell membranes, it is difficult for ADCs to target intracellular proteins, which makes it invalid for many tumor associated or specific proteins to be chosen as the targets of ADCs.

**Figure 1:**
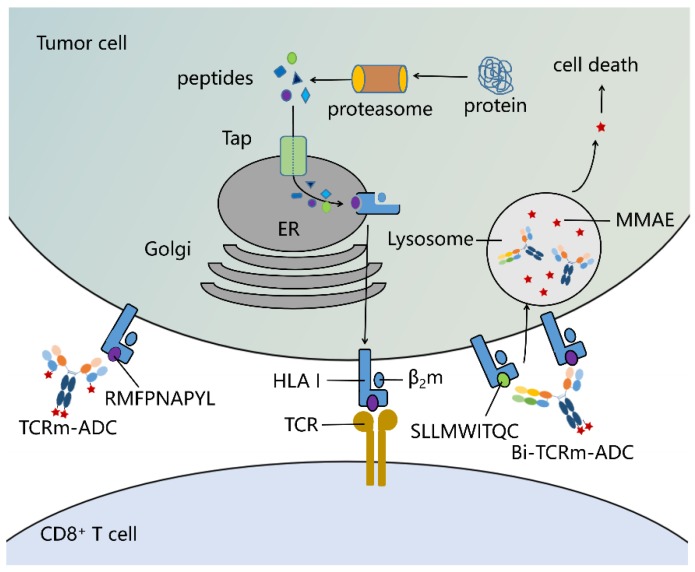
Peptide/human leukocyte antigen class I (HLA I) I complex as the targets of T cell receptor mimic antibody-drug conjugates (TCRm-ADCs) and bispecific(Bi)-TCRm-ADCs on the tumor cell surface. Peptides derived from intracellular proteins through proteasome hydrolysis are presented on the cell surface by HLA I molecules that can be specifically recognized by TCRs on T cells. TCRm-ADCs and Bi-TCRm-ADCs could be developed into T cell receptors (TCRs) binding via specifically recognizing peptide/HLA I complex. Therefore, TCRm-ADCs and Bi-TCRm-ADCs can specifically kill targeted tumor cells by delivering highly toxic agents (e.g., Monomethyl auristatin E (MMAE)) into cells after binding with the peptide/HLA I complex.

Intracellular proteins can be degraded, processed, and presented on the cell surface in a complex with major histocompatibility complex class I (MHC I) molecules, also known as human leukocyte antigen class I (HLA I) molecules in human beings, to form peptide/MHC complex (pMHC), which can be specifically recognized by T cell receptors (TCRs) (Figure 1) [4,5]. Antibodies targeting pMHCs like TCRs are called TCR-mimic antibodies (TCRm antibodies, also called TCR-like antibodies). At present, researchers have successfully developed high affinity TCRm antibodies against several tumor targets presented by different HLA alleles. The antitumor properties in preclinical models of TCRm antibodies have been confirmed, though the efficacy is not very satisfying [6,7,8,9,10]. Moreover, Lai et al. [11] and Lowe et al. [12] have proved that TCR-mimic antibody-drug conjugates (TCRm-ADCs) exhibit favorable antitumor effects in vitro and in vivo.

Wilms tumor 1 (WT1) oncoprotein is a transcription factor that is rarely expressed in normal adult tissues but overexpressed in a wide range of leukemia and solid tumors, particularly in mesothelioma, glioblastoma, gastrointestinal cancer, and ovarian cancer [13]. WT1 was ranked as one of the top cancer targets for immunotherapy by the National Institutes of Health-convened panel [14]. The 9-mer WT1-derived peptide 126–134, RMFPNAPYL (RMF), has been shown to be presented by HLA-A*02:01 molecules, which induces cytotoxic CD8^+^ T cells to kill WT1^+^ and HLA-A*02:01^+^ tumor cells [15,16,17]. Although several humanized TCRm antibodies targeting WT1 RMF/HLA-A*02:01 have been developed with high affinity and specificity (e.g., ESK1 and Q2L single-chain variable fragment-fragment crystallizable (scFv-Fc)), the potency was not ideal potentially due to extremely low epitope density (dozens to thousands) [8,18]. Over time, great efforts have been made to improve the efficacy of TCRm antibodies through affinity maturation, Fc glycosylation and chimeric antigen receptor(CAR) modification, which exhibited higher potency while none of them were applied in clinical studies [9,18,19]. Thus, it is necessary to find more powerful strategies to make up for the shortcomings of TCRm antibodies.

In this study, we established two TCRm-ADCs against WT1 RMF/HLA-A*02:01 complex with diverse binding kinetics parameters, which exhibited a moderate antitumor effect. And then we introduced bispecific antibody strategy to enhance the efficacy of TCRm-ADCs. Since there are no other antibodies targeting different epitopes of WT1 at present, we developed a bispecific (Bi)-TCRm-ADC against WT1 RMF/HLA-A*02:01 complex and NY-ESO-1 SLL/HLA-A*02:01 complex (Figure 1). NY-ESO-1 is a well-known cancer-testis antigen (CTA) re-expressed in numerous cancer types [20,21,22]. Both WT1 and NY-ESO-1 are highly expressed in esophageal carcinoma, lung squamous cell carcinoma, and rectum adenocarcinoma. The peptide SLLMWITQC (SLL, 157–165 amino acid derived from NY-ESO-1 protein) can be processed and presented on the surface of HLA-A*02:01 positive cells, which can trigger a cytotoxic T lymphocyte specific immune response [23]. The Bi-TCRm-ADC exerted more potent tumor cytotoxicity compared with TCRm-ADCs, which is a promising strategy to enhance the antitumor effects of TCRm-ADCs. Besides, several WT1 peptides had potential to be presented by HLA I molecules, which were predicted by NetMHC and NetCTL. Therefore, it is realistic to develop Bi-TCRm-ADCs targeting different pMHCs that are generated from one protein to improve the antitumor activity of pMHC targeted ADCs.

## 2. Results

### 2.1. Preparation and Characterization of TCRm Antibodies

**Figure 2:**
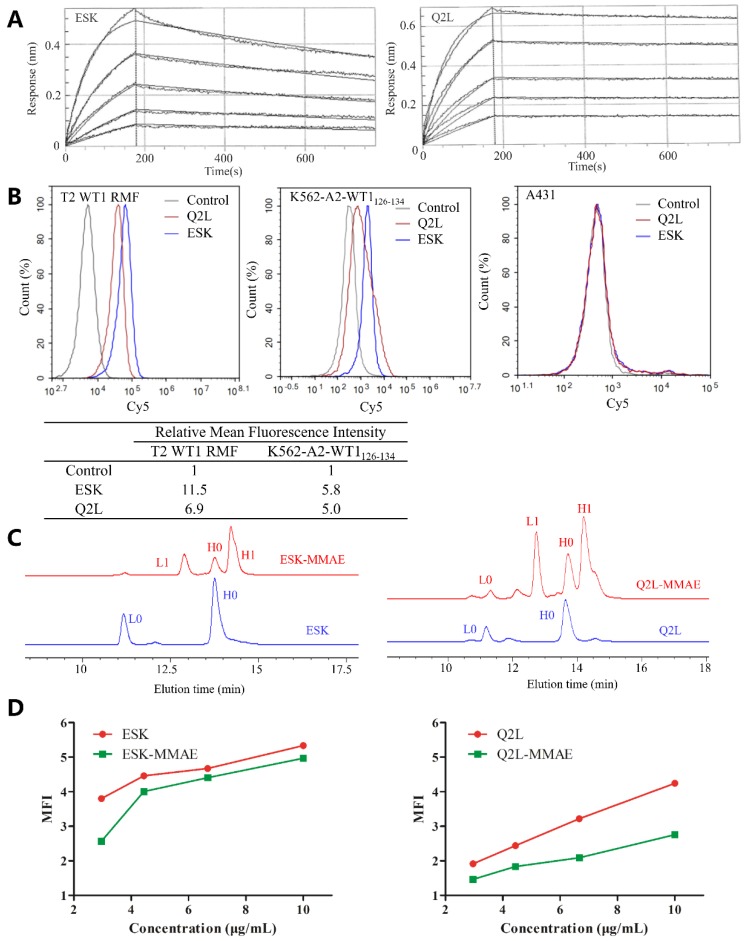
Characterization of TCRm antibodies and conjugates. (**A**) Sensorgrams of TCRm antibody in SPR analysis. (**B**) Binding of TCRm antibody to T2 plused with the WT1 RMF peptide, K562-A2-WT1_126–134_ and A431, and mean fluorescence intensity (MFI) was detected by flow cytometry. (**C**) RP-HPLC evaluation of sortase A-mediated conjugation between TCRm antibodies and vcMMAE. TCRm antibodies and their conjugates were reduced by dithiothreitol (DTT). L0 represented the light chain and H0 represented the heavy chain, while L1 indicated the light chain conjugated with vcMMAE and H1 indicated the heavy chain conjugated with vcMMAE. (**D**) Binding affinity of TCRm antibodies and their conjugates on K562-A2-WT1_126–134_ with serial concentrations.

ESK and Q2L, against WT1 RMF/HLA-A*02:01 complex, were successfully expressed by 293F cells and 293T cells, respectively. These antibodies were purified by protein A antibody affinity chromatography. The sensorgrams revealed differences in their dissociation rates (Figure 2A): The association constant (K_a_) and dissociation constant (K_d_) of ESK were higher than those of Q2L, indicating that ESK is easier to associate with and dissociate from WT1 RMF/HLA-A*02:01 complex compared with Q2L (Table 1). The binding affinity constant (K_D_) (K_d_/K_a_ ratio) values revealed that the affinity of ESK (3.50 nM) is higher than Q2L (9.09 nM).

T2 cell is an HLA-A*0201 positive and transporter-associated protein (TAP)-deficient cell line, there is no pMHC on its cell surface [24]. After β2 microglobulin (β2m) and peptide are exogenously added, specific pMHC can be formed on the surface of T2 cells [25]. K562-A2-WT1_126–134_ is a cell line we established to express HLA-A*02:01 and WT1 and the number of WT1 RMF/HLA-A*02:01 complex was 2.4 × 10^3^ copies per cell (Figure S2), while A431 is HLA-A*02:01^−^ and WT1^−^. We used RMF-pulsed T2, K562-A2-WT1_126–134_, and A431 to verify the specificity of ESK and Q2L. As depicted by Figure 2B, ESK and Q2L only recognized HLA-A*02:01^+^/WT1^+^ T2 and K562-A2-WT1_126–134_ and showed no non-specific binding with A431.

### 2.2. Sortase A Generated Site-Specific Conjugated TCRm-ADCs and Their Characterizations

After Sortase A mediated site-specific conjugation, ESK-MMAE and Q2L-MMAE were purified by protein A antibody affinity chromatography and analyzed by high-performance liquid chromatography (HPLC). The drug to antibody ratio (DAR) of ESK-MMAE and Q2L-MMAE was determined by reverse-phase high-performance liquid chromatography (RP-HPLC), which was calculated according to the peak-area ratio (DAR = (A_L1_/(A_L0_ + A_L1_) + A_H1_/(A_H0_ + A_H1_)) × 2). Interestingly, the DAR of both ESK-MMAE and Q2L-MMAE was 3.0 (Figure 2C). As Figure 2D showed, TCRm-ADCs could bind to K562-A2-WT1_126–134_ in a dose-dependent manner, which means that the higher the concentration is, the more TCRm-ADCs bind to the cell surface. The affinity of TCRm-ADCs was slightly attenuated after conjugation observed by flow cytometry.

### 2.3. Internalization of TCRm Antibodies and Their Conjugates

**Figure 3:**
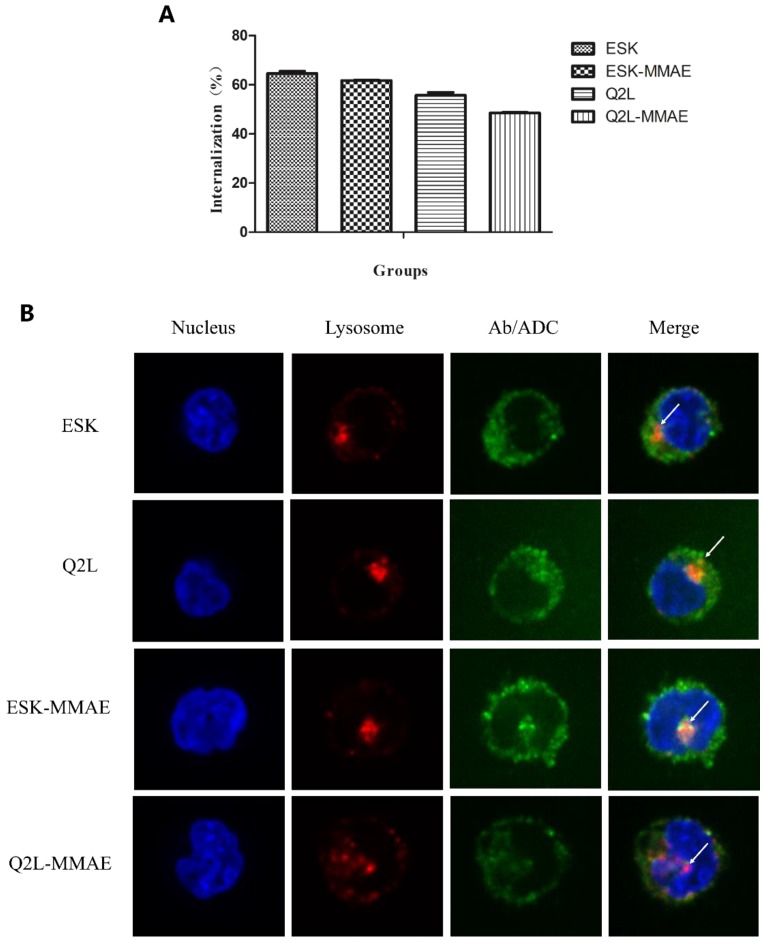
Cellular internalization of TCRm antibodies and their conjugates. (**A**) Cellular internalization ratio of TCRm antibodies and their conjugates determined by flow cytometry. Cellular internalization ratio (%) = (MFI of 4 °C − MFI of 37 °C)/MFI of 4 °C × 100%. (**B**) Cellular internalization of TCRm antibodies and their conjugates detected by fluorescence confocal microscope. Cells were imaged with DU-897D-CS0 rotary confocal laser scanning microscopy with 400× magnification. Blue fluorescent: DAPI, green fluorescent: Cy5, red fluorescent: Cy3, white arrows: TCRm antibodies and their conjugates co-located with lysosome.

Internalization is a predominant factor for ADCs to kill cancer cells after binding to antigen. Firstly, the flow cytometry was used to determine the internalization ratio of two TCRm antibodies and their conjugations. As shown in Figure 3A, approximately 50%–65% of TCRm antibodies and conjugates were internalized by K562-A2-WT1_126–134_ after 2 h of incubation. Internalization of ESK-MMAE and Q2L-MMAE was slightly declined after conjugation, which was consistent with the results of affinity evaluation. Furthermore, we verified the internalization of TCRm antibodies and conjugates by fluorescence confocal microscope. TCRm antibodies/TCRm-ADCs pre-incubated with K562-A2-WT1_126–134_ at 37 °C were stained with Cy5-labeled anti-human Fc antibody and lysosomal-associated membrane protein-1 (LAMP-1, the lysosomal marker) was stained by Cy3. As shown in Figure 3B, we found that both TCRm antibodies and TCRm-ADCs were observed in the cells, indicating that they could be all internalized by K562-A2-WT1_126–134_. Cy5 fluorescence and Cy3 fluorescence overlapped partly, indicating that TCRm antibodies and TCRm-ADCs can be transported into lysosomes for degradation to release MMAE molecules after they were internalized into cells.

### 2.4. In Vitro and In Vivo Antitumor Activity of TCRm-ADCs

**Figure 4:**
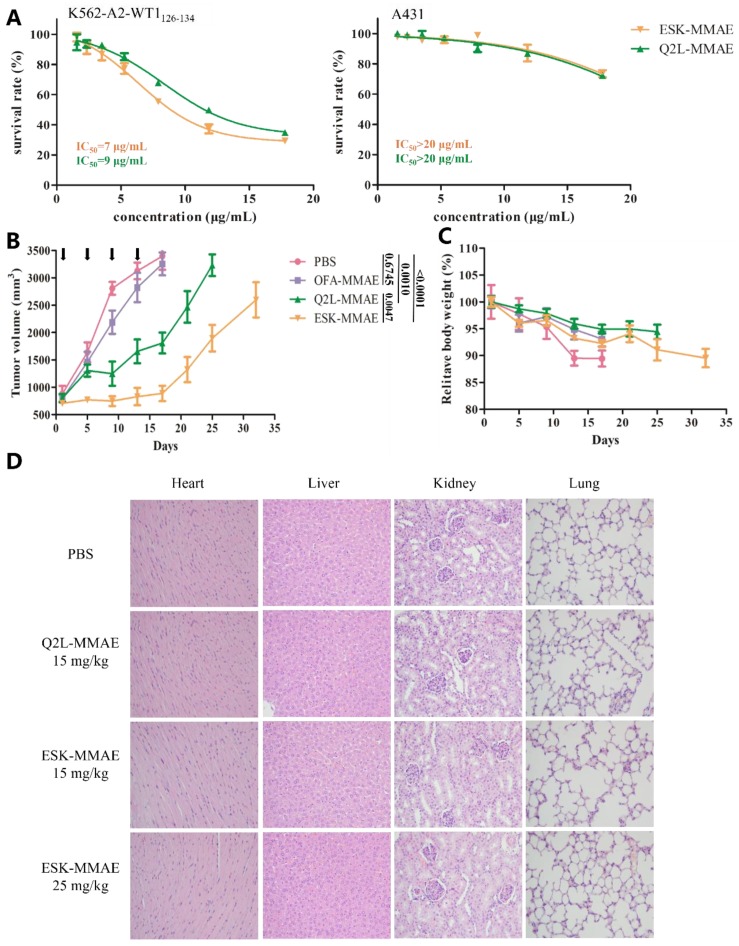
Antitumor activity and toxicity of TCRm-ADCs. (**A**) In vitro antitumor activity of Q2L-MMAE and ESK-MMAE on K562-A2-WT1_126–134_ (positive cell line) and A431 (negative cell line). (**B**) Antitumor activity of Q2L-MMAE and ESK-MMAE in K562-A2-WT1_126–134_ xenograft models (*n* = 5). Black arrow represents drug administration. *T*-test was used to determine the statistical significance (*p* value) among groups at day 17. (**C**) Relative body weight monitoring of mice after administration. (**D**) Systemic toxicity evaluation of primary organs under optical microscopy after hematoxylin and eosin (H&E) staining.

In order to evaluate the cytotoxicity of TCRm-ADCs, WT1 RMF/HLA-A*02:01 complex positive and negative cell lines were exposed to ESK-MMAE and Q2L-MMAE for 96 h. The results showed that ESK-MMAE and Q2L-MMAE displayed a specific killing effect on K562-A2-WT1_126–134_ compared to negative cells (A431) (Figure 4A). Although the affinity of ESK-MMAE was higher than that of Q2L-MMAE, two TCRm-ADCs showed similar antitumor activity on K562-A2-WT1_126–134_ and the IC_50_ of ESK-MMAE and Q2L-MMAE was 7 and 9 μg/mL, respectively, which was probably caused by the low peptide presentation on the K562-A2-WT1_126–134_ cell surface.

The in vivo efficacy of TCRm-ADCs was modeled from K562-A2-WT1_126–134_ leukemia xenograft model in BALB/c nude mice. Owing to the use of Matrigel, the tumor grew very fast. The mean tumor volume of the mice reached 700 mm^3^ (3.5–7 fold of tumor volume compared to the regular group which reached 100–200 mm^3^) [11,26] when we initiated administration. Anti-CD20 ADC (ofatumumab (OFA)-MMAE) was used as a negative control and was prepared at a DAR of 3.3 via a previous method [27]. As shown in Figure 4B, the mean tumor volume of PBS group and OFA-MMAE group (15 mg/kg) reached 3000 mm^3^ rapidly, and there was no significant difference of mean tumor volume between two groups (*p* = 0.6745). In contrast, Q2L-MMAE (15 mg/kg) and ESK-MMAE (15 mg/kg) were able to significantly delay tumor growth (*p* = 0.001 and *p* < 0.0001 compared with PBS group at day 17 respectively), which indicated that Q2L-MMAE and ESK-MMAE exhibited specific antitumor activity in vivo. The phenomenon that the tumors grew rapidly in TCRm-ADCs treated groups after stopping administration, implied that two TCRm-ADCs possessed the tumor inhibition effect. In addition, the antitumor activity of ESK-MMAE was significantly better than Q2L-MMAE (*p* = 0.0047 at day 17)

Meanwhile, we evaluated the in vivo toxicities of the TCRm-ADCs by monitoring the body weight (Figure 4C). Although the relative body weights of each group were slightly reduced, the body weight changes of TCRm-ADC-treated mice were less than that of PBS-treated mice, suggesting that the body weight of the mice was largely affected by tumor burden. For systemic toxicity evaluation, mice were sacrificed 10 days after administration, and histological sections of the major organs (heart, liver, kidney, and lung) of the mice were examined after hematoxylin and eosin (H&E) staining. No obvious histomorphologic changes were observed in any sections of organs (Figure 4D). Results all above illustrated that ESK-MMAE and Q2L-MMAE showed antitumor activity without obviously visible toxicity.

### 2.5. Preparation and Characterization of Bi-TCRm-ADC

**Figure 5:**
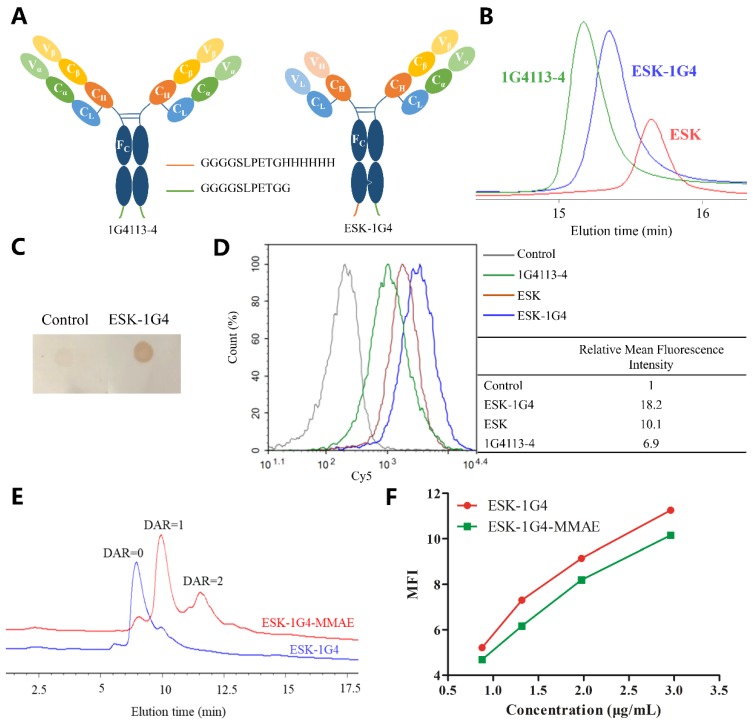
Preparation and characterization of ESK-1G4 and ESK-1G4-MMAE. (**A**) Schematic illustration of 1G4113-4 and ESK-1G4. (**B**) Analysis of 1G4113-4, ESK-1G4, and ESK via RP-HPLC under native condition. (**C**) Dot-ELISA to confirm the ability of ESK-1G4 to bind the two epitopes. (**D**) Binding affinity of ESK-1G4, ESK, and 1G4113-4 with K562-A2-NY-ESO-1_157–165_ by flow cytometry. (**E**) HIC analysis of the drug to antibody ratio (DAR) of ESK-1G4-MMAE under native condition. DAR 0, 1, or 2 means no, one or two vcMMAE molecules were conjugated to the intact antibody. (**F**) Binding affinity of ESK-1G4 and ESK-1G4-MMAE on K562-A2-NY-ESO-1_157–165_ with serial concentrations.

In order to enhance the efficacy of TCRm-ADCs, we used the “knobs-into-holes” approach to generate a hybrid IgG against WT1 RMF/HLA-A*02:01 complex and NY-ESO-1 SLL/HLA-A*02:01 complex (Figure 5A). After cotransfection of 293F cells with the “knob” and “hole” plasmids, we purified Bi-TCRm antibody by protein A antibody affinity chromatography and Ni-NTA affinity chromatography which assisted in the acquisition of pure and stable heterodimer (Figure 5B). The result of dot-ELISA showed that ESK-1G4 bound to both WT1 RMF/HLA-A*02:01-Fc complex and NY-ESO-1 SLL/HLA-A*02:01-Fc complex (Figure 5C). The results of flow cytometry manifested that the number of ESK-1G4 binding on K562-A2-NY-ESO-1_157–165_ was larger than that of ESK or 1G4113-4 (Figure 5D). K562-A2-NY-ESO-1_157–165_ is an HLA-A*02:01^+^, WT1^+^ and NY-ESO-1^+^ cell line and 1G4113-4 was a Fc-fused TCR against NY-ESO-1 SLL/HLA-A*02:01 complex that we established before (Figure 5A) [28]. To determine the DAR of ESK-1G4-MMAE, hydrophobic interaction chromatography (HIC) was used to separate intact antibodies. The results revealed that the DAR of ESK-1G4-MMAE was 1.2 (Figure 5E). And the affinity of ESK-1G4-MMAE was slightly attenuated after conjugation determined by flow cytometry (Figure 5F).

### 2.6. Internalization and In Vitro Cytotoxicity of Bi-TCRm-ADC

**Figure 6:**
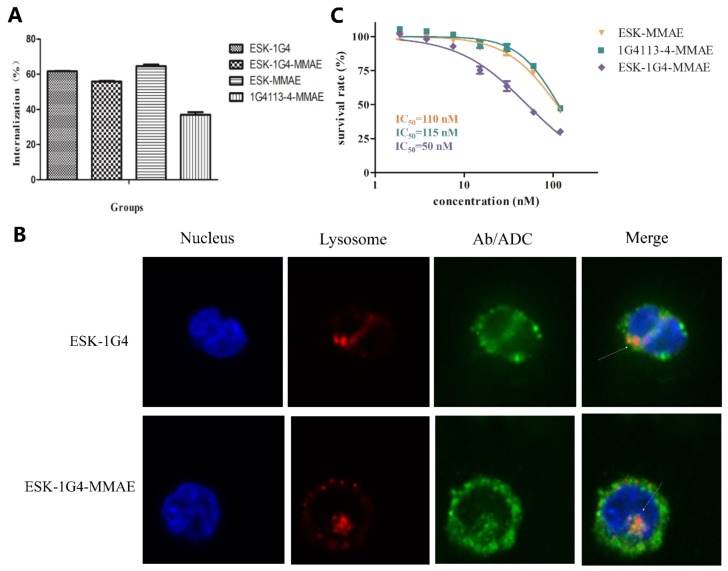
Internalization and in vitro cytotoxicity of ESK-1G4-MMAE (**A**) Cellular internalization ratio determined by flow cytometry. (**B**) Cellular internalization of ESK-1G4 and ESK-1G4-MMAE determined by fluorescence confocal microscope. Cells were imaged by DU-897D-CS0 rotary confocal laser scanning microscopy with 400× magnification. Blue fluorescent: DAPI, green fluorescent: Cy5, red fluorescent: Cy3, white arrows: TCRm antibodies or their conjugates located in lysosome. (**C**) In vitro antitumor activity on K562-A2-NY-ESO-1_157–165_. The X-axis represents the concentration of MMAE in the antibody-drug conjugates (ADCs)

The endocytic efficiency of ESK-1G4-MMAE was nice, a little bit lower than that of ESK-1G4 and ESK-MMAE, but much more higher than that of 1G4113-4-MMAE (Figure 6A). Moreover, subcellular trafficking and localization of ESK-1G4-MMAE in K562-A2-NY-ESO-1_157–165_ were further determined by fluorescence confocal microscope (Figure 6B). ESK-1G4-MMAE was within the cytoplasm after 4 h cell incubation, which showed the internalization. Furthermore, the internalized ESK-1G4-MMAE was co-localized with LAMP-1, indicating that ESK-1G4-MMAE could be transported into the lysosome after internalization to release the MMAE molecules.

We next determined the in vitro antitumor activity of ESK-1G4-MMAE, ESK-MMAE, and 1G4113-4-MMAE against K562-A2-NY-ESO-1_157–165_. ESK-1G4-MMAE showed more potency against K562-A2-NY-ESO-1_157–165_ than ESK-MMAE and 1G4113-4-MMAE (Figure 6C), suggesting that the increasing epitope density by constructing bispecific antibodies can effectively improve the antitumor activity of TCRm-ADCs.

### 2.7. Prediction of WT1 Protein Epitope Peptides

In search of potential presenting peptides of WT1 protein, the WT1 protein epitope peptides were predicted by online prediction algorithm. A total of 12 candidate peptides with the length of 8–11 derived from WT1 protein had suitable affinity to HLA-A*02:01 based on NetMHC binding prediction algorithm. The 12 peptides were subsequently validated in terms of cleavage sites of human proteasome and TAP transport efficiency in NetCTL. As shown in Table 2, except RMF peptide, other several peptides (e.g., ILCGAQYRI) not only with high affinity but also with high cleavage and TAP transport efficiency based on online service. Apart from HLA-A*02:01-restricted epitope peptides, there were several possible epitope peptides that have strong binding with other HLA alleles predicted by NetMHC (Table S1). These results suggested that in addition to RMF, the other WT1 protein peptides had potential to be presented by HLA I molecules as targets for tumor therapy which strengthens the advantages of our Bi-TCRm antibodies.

## 3. Discussion

ADCs, which exert antitumor activity through selectively tumor-targeting (derived from the antibody) and cytotoxicity (derived from the payload), are an attractive therapy used in clinical practice. However, one main problem is that ADCs in clinical mainly target cell surface proteins but are unable to access intracellular proteins. Considering that intracellular proteins can be degraded by proteasome, and these peptides derived from intracellular proteins could be presented by HLA-I. Herein, we described two sortase A-generated TCRm-ADCs (ESK-MMAE and Q2L-MMAE), against WT1 RMF/HLA-A*02:01 complex, which could specifically kill WT1 RMF/HLA-A*02:01 positive tumor cells in vitro (Figure 3C) and effectively inhibit tumor growth in the xenograft model (Figure 3D). Sortase A-mediated site-specific conjugation (C-terminal of the heavy chain and light chain) not only generates more homogeneous TCRm-ADCs, but also shows less loss of binding affinity comparing with assembling small-molecule drugs onto Lys or Cys residues of a tumor-targeting antibody randomly [29,30]. The higher antitumor potency of ESK-MMAE in the xenograft model compared with Q2L-MMAE is probably related to affinity and K_d_. Because antibodies with high affinity can effectively accumulate in tumor tissues and high K_d_ can efficient penetration into the large size tumor [31]. However, the size, charge, and shape of ADCs are also the key factors that affect antitumor efficiency, the definite influences of TCRm-ADCs’ efficacy need to be further studied.

The efficacy of the two TCRm-ADCs was unsatisfactory, which might largely depend on the fact that cell surface density of peptide/HLA I epitopes (dozens to thousands) is significantly lower than traditional antigens (tens of thousands to hundreds of thousands). Although chimeric antigen receptor T-cell immunotherapy (CAR-T) could enhance the efficacy of TCRm antibodies despite the very low density of the epitopes at the cell surface, CAR-T is so personalized that the cost is so high and so time-consuming that patients can’t afford to wait [19]. To date, extensive efforts have been made to raise the expression of peptide/HLA I epitopes to enhance the TCRm antibodies efficacy. Cytokines, such as IFN-γ and TNF-α, can promote the expression of HLA I and increase the expression of peptide/HLA I on the surface of tumor cells [32,33]. Lai et al. [11] confirmed that enhanced peptide presentation by trametinib, a MEK inhibitor, augmented the efficacy of TCRm-ADCs both in vitro and in vivo. In this study, we took another tack via using different peptide/HLA I epitopes to increase the number of targets, which is a supplement to the previous work and plays a synergistic role. Initially, we tried to construct Bi-TCRm antibody with ESK and Q2L, but there was a competitive binding between ESK and Q2L against WT1 RMF/HLA-A*02:01 complex (Figure S3). Therefore, we developed a Bi-TCRm-ADC (ESK-1G4-MMAE) against WT1 RMF/HLA-A*02:01 complex and NY-ESO-1 SLL/HLA-A*02:01 complex since the expression profiles of WT1 and NY-ESO-1 in tumor overlap partly, such as in esophageal carcinoma, lung squamous cell carcinoma, and rectum adenocarcinoma (Figure S4). Fortunately, the result is delightful that the efficacy of Bi-TCRm-ADC was apparently higher than that of TCRm-ADCs (Figure 6C). It indicates that increasing the number of targets by developing Bi-TCRm-ADCs is a potential effective way to enhance the potency of TCRm-ADCs. However, there are still many obstacles to the development of Bi-TCRm-ADCs. For example, it’s difficult to find two highly expressed tumor-specific proteins or tumor-associated proteins that presented by HLA I molecules on one natural tumor cell. We predicted the WT1 protein epitope peptides by NetMHC and NetCTL which showed that there were several peptides of WT1 presented by HLA I molecules in addition of RMF (Table 2 and Table S1) and it is corresponding to the prediction of NY-ESO-1 [34]. With the boom of antibody screening technologies, such as phage display technology [35] and yeast display technology [36], more and more TCRm antibodies will be identified against different epitope peptides from one protein. At that time, Bi- [37,38] and Tri- [38,39] TCRm-ADCs will improve the application of TCRm-ADCs.

HLA I-mediated presentation of intracellular tumor-associated proteins (including highly expressed proteins and mutant proteins) provides promising tumor targets for antibody-based immunotherapy (e.g., mAbs, ADCs, CAR-T, CAR-NK) and remarkably broadens antigen selection [11,18,19]. Tumor mutant peptides presented on the cell surface by HLA I molecules (e.g., KRAS G12V, EGFR L858R) as tumor-specific antigens (TSAs, also called neoantigens), are likely favorable targets for TCRm-ADCs since they are solely present on tumor cell surfaces, although it is still difficult so far for us to screen the specific TCRm antibodies which can discriminate between the wild types and mutant forms of the antigenic peptides, especially when some mutant residues are shielded by the HLA-I molecules [40]. Here we develop the TCRm-ADCs targeting tumor-associated antigen (TAA, WT1 RMF/HLA-A*02:01), which provides basis for the lucubration of TCRm-ADCs against mutant peptide/HLA, and contributes to precision medicine.

In summary, sortase A-generated TCRm-ADCs, ESK-MMAE, and Q2L-MMAE, against WT1 RMF/HLA-A*02:01 complex exhibited specific antitumor activity in vitro and in the xenograft model without obvious toxicity. Furthermore, the raise of Bi-TCRm-ADCs represents a promising strategy to enhance the antitumor effects of TCRm-ADCs. These new-generation TCRm-ADCs and Bi-TCRm-ADCs will broaden the utility of intracellular oncoproteins and ADCs.

## 4. Materials and Methods

### 4.1. Cell Culture

Human embryonic kidney cell 293F (American Type Culture Collection (ATCC), San Francisco, CA, USA) was cultured in shake flasks in 293-TI medium (Sino Biological Inc, Beijing, China) and 293T cell (ATCC) was cultured in DMEM medium (Invitrogen, Carlsbad, CA, USA) with 10% fetal bovine serum (FBS, Invitrogen). T2 cell was grown in IMDM (Invitrogen) supplemented with 20% FBS. Human epidermoid carcinoma cell line A431(ATCC) was grown in DMEM with 10% FBS. K562-A2 cell was constructed and maintained by our laboratory and was cultured in RPMI-1640 (Invitrogen) with 10% FBS. All cells were maintained in media with 100 U/mL penicillin (Solarbio, Beijing, China), 100 mg/mL streptomycin (Solarbio) at 37 °C with 5% CO_2_.

### 4.2. Antibodies and Sortase A Enzyme

The anti-WT1 RMF/HLA-A*02:01 scFvs were selected as previously described [8,18] and further engineered into a human IgG1 constant region to generate humanized antibodies named ESK and Q2L, respectively. The GGGGSLPETGG polypeptide sequence was introduced into the C-terminus of the light chain and heavy chain of the antibodies by PCR for subsequent enzymatic conjugation of sortase A as described previously [41]. TCRm antibodies were purified by protein A affinity chromatography (HiTrap Protein A HP column, GE Healthcare Life Sciences China, Beijing, China). The anti-NY-ESO-1 SLL/HLA-A*02:01 TCR (1G4113) was selected as previously described [42]. The approach of “knobs-into-holes” was used to generate a hybrid IgG (ESK-1G4) against WT1 RMF/HLA-A*02:01 and NY-ESO-1 SLL/HLA-A*02:01 [43]. The C-terminus of the heavy chain of ESK and 1G4113-4 was genetically fused with GGGGSLPETGHHHHHH and GGGGSLPETGG, respectively. ESK-1G4 was purified by protein A antibody affinity chromatography and Ni-NTA affinity chromatography (HisTrap HP column, GE Healthcare Life Sciences China). Wild sortase A enzymes were expressed and purified as described previously [29].

### 4.3. Surface Plasmon Resonance (SPR) Analysis

Binding kinetics parameters were performed on a Fortebio Octet RED384 instrument (Pall Corporation, New York, NY, USA) using an anti-human Fab-CH1 2nd Generation (FAB2G) Biosensor chip as previously described [44]. Briefly, TCRm antibodies were prepared at 30 µg/mL in 1× KB running buffer and dispensed into a 384-well tilted-bottom microplate (90 µL per well). Another 384-well microplate contained WT1 RMF/HLA-A*02:01-Fc complex at 5 different concentrations (6.25–100 nM (ESK)/100–1600 nM (Q2L), in 2-fold serial dilutions). Binding kinetics were measured by dipping the TCRm antibodies coated sensor in wells containing various concentrations of the WT1 RMF / HLA-A*02:01-Fc complex.

### 4.4. Dot-ELISA for Verifying the Expression of ESK-1G4

NY-ESO-1 SLL/HLA-A*02:01-Fc (Supplementary Materials) was spotted on a nitrocellulose membrane (Beyotime, Shanghai, China). The nitrocellulose membrane was immersed in a dot-ELISA blocking buffer (PBS buffer with 0.1% Tween 20 and 10% skimmed milk) and blocked at 37 °C for 1 h after dried. Ten μg/mL ESK-1G4 was diluted in dot-ELISA blocking buffer and incubated with the nitrocellulose membrane for 1 h at 37 °C. After the incubation, the nitrocellulose membrane was washed 4 times with a dot-ELISA washing buffer (PBS buffer with 0.1% Tween 20). The nitrocellulose membrane was then incubated with WT1 RMF/HLA-A*02:01-Fc-biotin at 37 °C for 1 h and washed as above. The HRP-labeled streptavidin was then incubated with the nitrocellulose membrane for 1 h at 37 °C followed by washing as above. Finally, the nitrocellulose membrane was put in a DAB horseradish peroxidase coloring buffer at room temperature.

### 4.5. Sortase A-Mediated Conjugation of Antibodies with Gly_3_-Val-Cit-PAB-MMAE

Antibodies with C-terminal LPETG sequence were conjugated to Gly_3_-val-cit-PAB-MMAE (vcMMAE, Concortis, San Diego, CA, USA) by incubating 2 μM antibodies with 200 μM vcMMAE in the presence of 50 μM sortase A in reaction buffer (50 mM Tris, 150 mM NaCl, 5 mM CaCl_2_, pH 7.5) for 24 h at 37 °C. ADCs were purified by protein A chromatography and washed repeatedly with PBS by ultrafiltration.

### 4.6. Characterization of ADCs

RP-HPLC was performed using the Agilent PLRP-S column (100 Å, 8 µm, 2.1 × 150 mm). Elution buffer A was 0.1% trifluoroacetic acid in ultrapure water and elution buffer B was acetonitrile. The conditions were as follows: Column temperature: 50 °C; Gradient elution: 0–3 min 25% B, 3–20 min 25–50% B, 20–22 min 50–95% B; 22–24 min 95–25% B; 24–26 min 25% B at 0.6 mL/min. TCRm antibodies and conjugates were previously reduced by DTT at 37 °C for 2 h and filtered (0.22 µm) or centrifuged (12,000 g, 30 min) to remove precipitants before performing RP-HPLC.

HIC was performed on a TOSOH Butyl-NPR column (2.5 µm, 4.6 mm × 3.5 cm) at 0.6 mL/min with a 15 min linear gradient elution from 1.5 M (NH_4_)_2_SO_4_ and 25 mM Na_3_PO_4_ (pH 7.0) to 25 mM Na_3_PO_4_ (pH = 7.0) and 25% isopropanol.

### 4.7. Flow Cytometry Analysis for Cell-Binding Assays

#### 4.7.1. Binding of TCRm Antibodies on Different Cell Lines

T2 is a mutant cell line that lacks TAP which allows for efficient loading of exogenous peptides. The 5 × 10^5^ T2 cells were cultured in serum-free IMDM medium containing 25 μg/mL RMF peptide and 5 μg/mL human β2m (Sigma-Aldrich, St. Louis, MO, USA) overnight at 37 °C, 5% CO_2_. Then, cells were washed with ice-cold staining buffer (1% BSA in PBS) and incubated with 10 μg/mL ESK or Q2L for 30 min on ice. After staining buffer washing, cells were incubated with secondary Cy5-labeled Goat Anti-Human IgG (H+L) polyclonal antibody (Abcam, Cambridge, UK) and the MFI was measured on ACEA NovoCyteTM flow cytometry.

The 5 × 10^5^ K562-A2-WT1_126–134_ cells (Supplementary Materials) or A431 cells were collected and incubated with 10 μg/mL ESK or Q2L and then immunostained and analyzed by flow cytometry as above.

#### 4.7.2. Influence of Binding Affinity of ESK, Q2L, and ESK-1G4 after Sortase A-Mediated Conjugation on K562-A2-WT1_126–134_ Cell Line

The 5 × 10^5^ K562-A2-WT1_126–134_ cells were incubated with serial concentrations of ESK, Q2L, ESK-1G4, and their conjugates in PBS on ice for 30 min. Then the cells were washed, immunostained, and analyzed as above.

### 4.8. Cellular Internalization

#### 4.8.1. Cellular Internalization Ratio of TCRm Antibodies and Their Conjugates

K562-A2-WT1_126–134_ were incubated with 10 μg/mL TCRm antibodies or their conjugates on ice for 30 min and then washed twice with PBS. Cells were incubated at 37 or 4 °C for 2 h, then washed by PBS and stained with Cy5-labeled Goat Anti-Human IgG (H+L) polyclonal antibody (Abcam). The MFI was measured and analyzed as above. Cellular internalization ratio of TCRm antibodies and conjugates was determined by the following formula: Cellular internalization ratio (%) = (MFI of 4 °C − MFI of 37 °C)/MFI of 4 °C × 100%.

#### 4.8.2. Microscopy for TCRm Antibodies and Conjugates Trafficking

The 2 × 10^4^ K562-A2-WT1_126–134_ cells were seeded on slides and treated with TCRm antibody or its conjugates at 37 °C for 6 h. The supernatant was discarded and the cells were gently washed twice with PBS. Cells were fixed with 4% paraformaldehyde for 15 min. After washed with PBS, cells were permeabilized with 0.1% Triton X-100, 0.2% BSA in PBS for 10 min, followed by blocking with 2% BSA-PBS for 30 min. Then, cells were incubated with rabbit anti-human lysosome antibody (Abcam, Cambridge, UK) in 1% BSA-PBS for 45 min. After gently washed with PBS, cells were stained with Cy3-labeled Goat Anti-Rabbit IgG (H+L) polyclonal antibody (Beyotime, Shanghai, China) and Cy5-labeled Goat Anti-Human IgG (H+L) polyclonal antibody (Abcam, Cambridge, UK for 45 min. After washing, nuclei were further stained with DAPI (Beyotime, Shanghai, China) for 3 min and the excess dye was washed off. Then, cells were covered with coverslip. Fluorescence images were acquired by DU-897D-CS0 rotary confocal laser scanning microscopy.

### 4.9. In Vitro Efficacy

The cytotoxicity of ESK-MMAE and Q2L-MMAE was assessed on K562-A2-WT1_126–134_ and A431 cell lines. Briefly, 3000 cells were seeded in 96-well plates and incubated with different concentrations of ESK-MMAE and Q2L-MMAE (3 replicates per concentration, 0 μg/mL was the control group) at 37 °C, 5% CO_2_ for 96 h. After incubation, cells were incubated with 10% Cell Counting Kit-8 (CCK-8) (Dojindo, Kumamoto, Japan) for 2–3 h, and then the OD_450_ value was measured using a BioRad Model 680 Microplate Reader. According to the formula: The survival rate = the OD_450_ value of different concentration wells/the average value of OD_450_ of the control group × 100%, the survival rate of each well cell was calculated. The IC_50_, which represents the concentration of a drug that is required for 50% inhibition in vitro, was calculated by GraphPad Prim 6.01 software (Graphpad, San Diego, CA, USA based on the survival rate.

### 4.10. Mouse Xenograft Study

Six to eight-week-old male BALB/c nude mice were purchased from (SHANGHAI SLAC Shanghai, China) and were housed in a specific pathogen free facility. Mice were pretreated by intraperitoneal injections of cyclophosphamide once a day at a dose of 50 mg/kg for two days. Two days after the second cyclophosphamide injection, approximately 1 × 10^7^ K562-A2-WT1_126–134_ cells in 100 µL PBS and 100 µL Matrigel were inoculated subcutaneously into the right flank of nude mice. Day 8 after inoculation, the mice were randomly divided into 4 groups (with 5 mice per group): PBS, OFA-MMAE (15 mg/kg), Q2L-MMAE (15 mg/kg), and ESK-MMAE (15 mg/kg). Each group was treated every four days for four times (q4d × 4) via the tail vein. The body weight and tumor volume (V = (L × W^2^)/2, L is length and W is width of tumor) were monitored every 4 days until the end of the experiment. All animal experiments were performed in accordance with the National Institute of Health Guide for the Care and Use of Laboratory Animals. The protocols were approved by the Committee on the Ethics of Animal Experiments of the Zhejiang University, China (ZJU20170435, the date of approval: 20170428).

The possible toxicity in heart, liver, kidney, or spleen was examined 10 days after administration by H&E staining.

Statistical Analysis: T-test was used to determine statistical significance, *p* < 0.05 was considered statistically significant.

### 4.11. Prediction of WT1 Protein Epitope Peptides

Amino acid sequence of WT1 protein was applied to the HLA-binding prediction algorithm, NetMHC (Available online: http://www.cbs.dtu.dk/services/NetMHC/). Intermediate binding affinity (IC_50_ ≤ 500 nM) with HLA-A*02:01 allele was set as threshold to screen epitope peptides. Then, the candidate epitope peptides were assessed based on cleavage sites of human proteasome and TAP transport efficiency by NetCTL (Awailable online: http://www.cbs.dtu.dk/services/NetCTL/). At last, we summarized the WT1 protein epitope peptides restricted to HLA-A*02:01 allele after comprehensive analysis.

## Figures and Tables

**Table 1:** Affinity of ESK and Q2L.

TCRm Antibodies	IgG Isotype	K_a_ (1/Ms)	K_d_ (1/s)	K_D_ (nM)
ESK	Hu IgG1	1.64 × 10^5^	5.73 × 10^−4^	3.50
Q2L	Hu IgG1	9.96 × 10^3^	9.03 × 10^−5^	9.09

**Table 2:** Comprehensive analysis of WT1 protein HLA-A*02:01-restricted epitope peptides by different online prediction tools.

Peptide Length	Position	Peptide Sequence	NetCTL		NetMHC
			Cleavage	Tap	Affinity(nM)
8	8–15	LNALLPAV	0.482568	NK	274.80
9	10–18	ALLPAVPSL	0.028869	1.2770	6.01
	126–134	RMFPNAPYL	0.244498	1.5860	7.14
	187–195	SLGEQQYSV	0.143282	0.1940	21.76
	191–200	QQYSVPPPV	0.044789	0.4230	29.84
	225–233	NLYQMTSQL	0.090656	1.0590	236.39
	280–288	ILCGAQYRI	0.308379	0.4410	128.47
10	6–15	RDLNALLPAV	0.093866	NK	33.75
	9–18	RMFPNAPYLP	0.802949	NK	173.54
	125–134	ARMFPNAPYL	0.297255	NK	28.46
	126–135	ALLPAVPSLG	0.035614	NK	223.13
11	5–15	VRDLNALLPAV	0.018917	NK	285.66

Position: Position of Peptide. Cleavage: C-terminal cleavage affinity. Tap: TAP transport efficiency. Affinity: Predicted HLA binding affinity. NK: Not know.

## Supplementary Materials

Supplementary materials can be found at https://www.mdpi.com/1422-0067/20/16/3912/s1.

## References

[1] de Goeij B.E., Lambert J.M. (2016). New developments for antibody-drug conjugate-based therapeutic approaches. Curr. Opin. Immunol..

[2] Weiner L.M., Murray J.C., Shuptrine C.W. (2012). Antibody-based immunotherapy of cancer: New insights, new targets. Cell.

[3] Scott A.M., Wolchok J.D., Old L.J. (2012). Antibody therapy of cancer. Nat. Rev. Cancer.

[4] York I.A., Rock K.L. (1996). Antigen processing and presentation by the class I major histocompatibility complex. Annu. Rev. Immunol..

[5] Vyas J.M., Van Der Veen A.G., Ploegh H.L. (2008). The known unknowns of antigen processing and presentation. Nat. Rev. Immunol..

[6] Epel M., Carmi I., Soueid-Baumgarten S., Oh S.K., Bera T., Pastan I., Berzofsky J., Reiter Y. (2008). Targeting TARP, a novel breast and prostate tumor-associated antigen, with T cell receptor-like human recombinant antibodies. Eur. J. Immunol..

[7] Jain R., Rawat A., Verma B., Markiewski M.M., Weidanz J.A. (2013). Antitumor activity of a monoclonal antibody targeting major histocompatibility complex class I-Her2 peptide complexes. J. Natl. Cancer Inst..

[8] Dao T., Yan S., Veomett N., Pankov D., Zhou L., Korontsvit T., Scott A., Whitten J., Maslak P., Casey E. (2013). Targeting the intracellular WT1 oncogene product with a therapeutic human antibody. Sci. Transl. Med..

[9] Veomett N., Dao T., Liu H., Xiang J., Pankov D., Dubrovsky L., Whitten J.A., Park S.M., Korontsvit T., Zakhaleva V. (2014). Therapeutic efficacy of an Fc-enhanced TCR-like antibody to the intracellular WT1 oncoprotein. Clin. Cancer Res..

[10] Chang A.Y., Dao T., Gejman R.S., Jarvis C.A., Scott A., Dubrovsky L., Mathias M.D., Korontsvit T., Zakhaleva V., Curcio M. (2017). A therapeutic T cell receptor mimic antibody targets tumor-associated PRAME peptide/HLA-I antigens. J. Clin. Invest..

[11] Lai J., Wang Y., Wu S.S., Ding D., Sun Z.Y., Zhang Y., Zhou J., Zhou Z., Xu Y.C., Pan L.Q. (2018). Elimination of melanoma by sortase A-generated TCR-like antibody-drug conjugates (TL-ADCs) targeting intracellular melanoma antigen MART-1. Biomaterials.

[12] Lowe D.B., Bivens C.K., Mobley A.S., Herrera C.E., McCormick A.L., Wichner T., Sabnani M.K., Wood L.M., Weidanz J.A. (2017). TCR-like antibody drug conjugates mediate killing of tumor cells with low peptide/HLA targets. MAbs.

[13] Keilholz U., Menssen H.D., Gaiger A., Menke A., Oji Y., Oka Y., Scheibenbogen C., Stauss H., Thiel E., Sugiyama H. (2005). Wilms’ tumour gene 1 (WT1) in human neoplasia. Leukemia.

[14] Cheever M.A., Allison J.P., Ferris A.S., Finn O.J., Hastings B.M., Hecht T.T., Mellman I., Prindiville S.A., Viner J.L., Weiner L.M. (2009). The prioritization of cancer antigens: A National Cancer Institute pilot project for the acceleration of translational research. Clin. Cancer Res..

[15] Keilholz U., Letsch A., Busse A., Asemissen A.M., Bauer S., Blau I.W., Hofmann W.-K., Uharek L., Thiel E., Scheibenbogen C. (2009). A clinical and immunologic phase 2 trial of Wilms tumor gene product 1 (WT1) peptide vaccination in patients with AML and MDS. Blood.

[16] Rezvani K., Yong A.S.M., Mielke S., Savani B.N., Musse L., Superata J., Jafarpour B., Boss C., Barrett A.J. (2008). Leukemia-associated antigen-specific T-cell responses following combined PR1 and WT1 peptide vaccination in patients with myeloid malignancies. Blood.

[17] Krug L.M., Dao T., Brown A.B., Maslak P., Travis W., Bekele S., Korontsvit T., Zakhaleva V., Wolchok J., Yuan J. (2010). WT1 peptide vaccinations induce CD4 and CD8 T cell immune responses in patients with mesothelioma and non-small cell lung cancer. Cancer Immunol. Immunother..

[18] Zhao Q., Ahmed M., Tassev D.V., Hasan A., Kuo T.-Y., Guo H.-F., O’Reilly R.J., Cheung N.-K.V. (2015). Affinity maturation of T-cell receptor-like antibodies for Wilms tumor 1 peptide greatly enhances therapeutic potential. Leukemia.

[19] Rafiq S., Purdon T.J., Daniyan A.F., Koneru M., Dao T., Liu C., Scheinberg D.A., Brentjens R.J. (2017). Optimized T-cell receptor-mimic chimeric antigen receptor T cells directed toward the intracellular Wilms Tumor 1 antigen. Leukemia.

[20] Akcakanat A., Kanda T., Koyama Y., Watanabe M., Kimura E., Yoshida Y., Komukai S., Nakagawa S., Odani S., Fujii H. (2004). NY-ESO-1 expression and its serum immunoreactivity in esophageal cancer. Cancer Chemother. Pharm..

[21] Prasad M.L., Jungbluth A.A., Patel S.G., Iversen K., Hoshaw-Woodard S., Busam K.J. (2004). Expression and significance of cancer testis antigens in primary mucosal melanoma of the head and neck. Head Neck.

[22] Thomas R., Al-Khadairi G., Roelands J., Hendrickx W., Dermime S., Bedognetti D., Decock J. (2018). NY-ESO-1 based immunotherapy of cancer: Current perspectives. Front. Immunol..

[23] McCormack E., Adams K.J., Hassan N.J., Kotian A., Lissin N.M., Sami M., Mujić M., Osdal T., Gjertsen B.T., Baker D. (2013). Bi-specific TCR-anti CD3 redirected T-cell targeting of NY-ESO-1- and LAGE-1-positive tumors. Cancer Immunol. Immunother..

[24] Salter R.D., Howell D.N., Cresswell P. (1985). Genes regulating HLA class I antigen expression in T-B lymphoblast hybrids. Immunogenetics.

[25] Brickner A.G. (2006). The PANE1 gene encodes a novel human minor histocompatibility antigen that is selectively expressed in B-lymphoid cells and B-CLL. Blood.

[26] Li Z.H., Zhang Q., Wang H.B., Zhang Y.N., Ding D., Pan L.Q., Miao D., Xu S., Zhang C., Luo P.H. (2014). Preclinical studies of targeted therapies for CD20-positive B lymphoid malignancies by Ofatumumab conjugated with auristatin. Invest. New Drugs.

[27] Xu Y., Jin S., Zhao W., Liu W., Ding D., Zhou J., Chen S. (2017). A versatile chemo-enzymatic conjugation approach yields homogeneous and highly potent antibody-drug conjugates. Int. J. Mol. Sci..

[28] Zhao W. (2019). Study on the Factors of Safety and Efficacy of Novel Antibody-Drug Conjugates. Ph.D. Thesis.

[29] Pan L., Zhao W., Lai J., Ding D., Zhang Q., Yang X., Huang M., Jin S., Xu Y., Zeng S. (2017). Sortase A-generated highly potent anti-CD20-MMAE conjugates for efficient elimination of B-lineage lymphomas. Small.

[30] Wakankar A., Chen Y., Gokarn Y., Jacobson F.S. (2011). Analytical methods for physicochemical characterization of antibody drug conjugates. MAbs.

[31] Zuckier L.S., Berkowitz E.Z., Sattenberg R.J., Zhao Q.H., Deng H.F., Scharff M.D. (2000). Influence of affinity and antigen density on antibody localization in a modifiable tumor targeting model. Cancer Res..

[32] Zhou F. (2009). Molecular mechanisms of IFN-γ to up-regulate MHC class I antigen processing and presentation. Int. Rev. Immunol..

[33] Yang Y.M., Shang D.S., Zhao W.D., Fang W.G., Chen Y.H. (2013). Microglial TNF-α-dependent elevation of MHC class I expression on brain endothelium induced by amyloid-beta promotes T cell transendothelial migration. Neurochem. Res..

[34] Jäger E., Chen Y.-T., Drijfhout J.W., Karbach J., Ringhoffer M., Jäger D., Arand M., Wada H., Noguchi Y., Stockert E. (2002). Simultaneous humoral and cellular immune response against cancer–testis antigen NY-ESO-1: Definition of human histocompatibility leukocyte antigen (HLA)-A2–binding peptide epitopes. J. Exp. Med..

[35] Ledsgaard L., Kilstrup M., Karatt-Vellatt A., McCafferty J., Laustsen A.H. (2018). Basics of antibody phage display technology. Toxins.

[36] Tillotson B.J., Lajoie J.M., Shusta E.V. (2015). Yeast display-based antibody affinity maturation using detergent-solubilized cell lysates. Methods Mol. Biol..

[37] Brinkmann U., Kontermann R.E. (2017). The making of bispecific antibodies. MAbs.

[38] Runcie K., Budman D.R., John V., Seetharamu N. (2018). Bi-specific and tri-specific antibodies- the next big thing in solid tumor therapeutics. Mol. Med..

[39] Steinhardt J.J., Guenaga J., Turner H.L., McKee K., Louder M.K., O’Dell S., Chiang C.I., Lei L., Galkin A., Andrianov A.K. (2018). Rational design of a trispecific antibody targeting the HIV-1 Env with elevated anti-viral activity. Nat. Commun..

[40] Skora A.D., Douglass J., Hwang M.S., Tam A.J., Blosser R.L., Gabelli S.B., Cao J., Diaz L.A., Papadopoulos N., Kinzler K.W. (2015). Generation of MANAbodies specific to HLA-restricted epitopes encoded by somatically mutated genes. Proc. Natl. Acad. Sci..

[41] Beerli R.R., Hell T., Merkel A.S., Grawunder U. (2015). Sortase enzyme-mediated generation of site-specifically conjugated antibody drug conjugates with high in vitro and in vivo potency. PloS ONE.

[42] Li Y., Moysey R., Molloy P.E., Vuidepot A.L., Mahon T., Baston E., Dunn S., Liddy N., Jacob J., Jakobsen B.K. (2005). Directed evolution of human T-cell receptors with picomolar affinities by phage display. Nat. Biotechnol..

[43] Chen C., Zhang Y., Zhang Y., Li J., Tsao S.W., Zhang M.-Y. (2013). Superior antitumor activity of a novel bispecific antibody cotargeting human epidermal growth factor receptor 2 and type I insulin-like growth factor receptor. Mol. Cancer.

[44] Yang D., Singh A., Wu H., Kroe-Barrett R. (2016). Dataset of the binding kinetic rate constants of anti-PCSK9 antibodies obtained using the Biacore T100, ProteOn XPR36, Octet RED384, and IBIS MX96 biosensor platforms. Data Br..

