# Supplementary material for: The Antitumor Activity of TCR-Mimic Antibody-Drug Conjugates (TCRm-ADCs) Targeting the Intracellular Wilms Tumor 1 (WT1) Oncoprotein
Source: Int J Mol Sci. 2019 Aug 12;20(16):3912. doi: 10.3390/ijms20163912 (PMC6720711; doi:10.3390/ijms20163912)
Supplement: Supplementary file 1 [file ijms-20-03912-s001.zip › ijms-552183-supplementary.docx]

1. NY-ESO-1 SLL/HLA-A*02:01-Fc and WT1 RMF/HLA-A*02:01-Fc-Biotin

Recombinant peptide/HLA-A*02:01-Fc fusion (the C-terminus of the Fc including GGGGSLPETGG sequence) was cloned into standard mammalian expression vectors pcDNA3.1-hygro(+) and transiently transfected into 293F cells. Fusion proteins were purified from the supernatant by protein A affinity chromatography. Purified proteins were analyzed by SDS-PAGE (Figure S1) and ELISA. And then, 2 μμM WT1 RMF/HLA-A*02:01-Fc and 200 μM GGGK-biotin (Bankpeptide) were conjugated by 50 μM sortase A in Tris-NaCl buffer which contained 5 mM CaCl_2_. WT1 RMF/HLA-A*02:01-Fc-biotin was purified by protein A affinity chromatography and verified via ELISA with streptavidin.

1. K562-A2-WT1126-134 Cell Model

Fusion expression of ubiquitin (Ub) and RMF peptide was used to construct the cell line with relatively high expression of the WT1 RMF/HLA-A*02:01 complex. The *Eco*R I-EGFP-Ub-WT1-RMF-Not I gene was synthesized by Sangon Biotech (Shanghai, China). The gene and pMH3 vector were digested with *Eco*R I (Takara) and Not I (Takara), and then ligated with T4 ligase (Takara) and transfected with DH5α competent state. The monoclonal colonies were picked for sequencing verification, and the correct clones were used to extract endotoxin-free plasmids (EndoFree Maxi Plasmid Kit, TIANGEN). The plasmid was transfected into K562-A2 cells by lipofectamine 3000 (Invitrogen). The next day after transfection, the previous medium was discarded and fresh medium containing 800 μg/mL G418 sulfate (BBI Life Sciences) was added. After G418-resistant cells were overgrown in culture vessels, about 100 cells were taken and evenly distributed into wells in a 96-well cell culture plate. EGFP green fluorescent signal and ESK were used to screen monoclonal cells with stable expression of EGFP-Ub-RMF by flow cytometry.

1. Evaluation the Amount of WT1 RMF/HLA-A*02:01 Complex Presentation on the Surface of K562-A2-WT1126-134 Cell Line

We used flow cytometry to determine the corresponding mean fluorescence intensity (MFI of the magnetic beads labeled with a specific number of PE molecules (BD Quantibrite™ PE Beads) and drawn a standard curve as shown in Figure S2A. The PE molecule was labeled on the ESK (1:1) by SiteClick™ Antibody Labeling Kits (Invitrigen). The 5 × 10^5^ K562-A2-WT1_126-134_ cells were collected and incubated with 10 μg/mL ESK-PE and analyzed by flow cytometry (Figure S2B). Based on the measured MFI and standard curve, the number of WT1 RMF/HLA-A*02:01 complex on the surface of K562-A2-WT1_126-134_ cells was calculated to be 2.4 × 10^3^ copies per cell.

1. Competition-Binding Assays

To evaluate the competition-binding of ESK and Q2L, 5 × 10^5^ K562-A2-WT1_126-134_ cells in triplicate were incubated with 10 μg/mL ESK (Q2L), after PBS washing, cells were incubated with 10 μg/mL Q2L (ESK). At the same time, another 5 × 10^5^ K562-A2-WT1_126-134_ cells in triplicate were incubated with 10 μg/mL ESK or Q2L. Then the cells were washed, immunostained, and analyzed by flow cytometry. As Figure S3 showed, the MFI of cells incubated with both ESK and Q2L didn’t increase compared with that of cells incubated with ESK, suggesting there is competition between ESK and Q2L binding on WT1 RMF/HLA-A*02:01 complex.


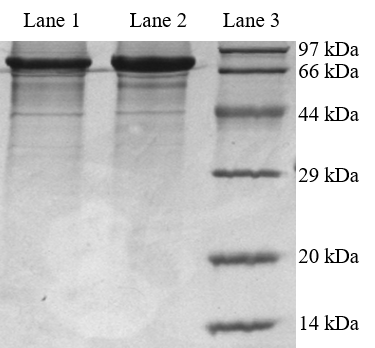


**
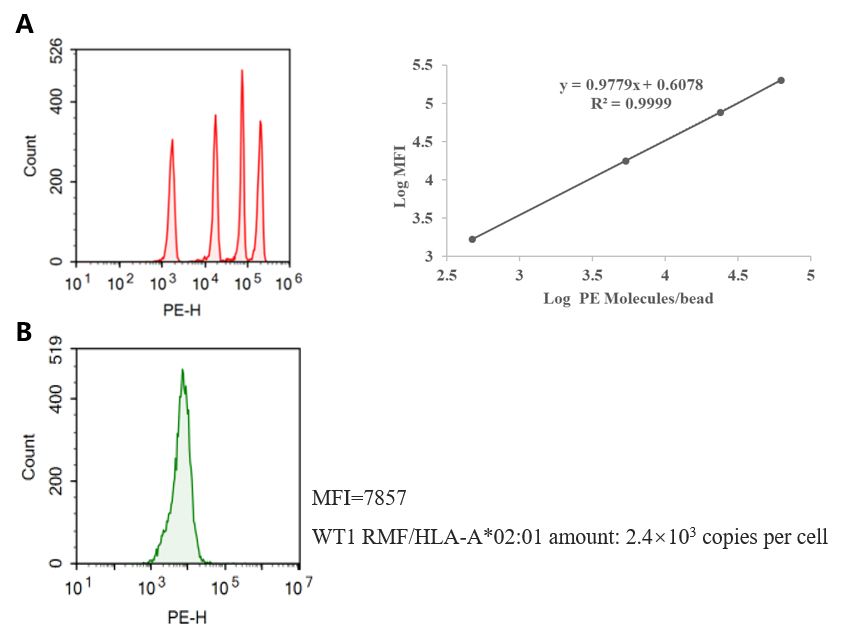
Figure S1.** Analysis of NY-ESO-1 SLL/HLA-A*02:01-Fc and WT1 RMF/HLA-A*02:01-Fc by SDS-PAGE. Lane 1: NY-ESO-1 SLL/HLA-A*02:01-Fc; Lane 2: WT1 RMF/HLA-A*02:01-Fc, Lane 3: Protein mark.

**Figure S2.** Evaluation the amount of WT1 RMF/HLA-A*02:01 complex presentation on the surface of the K562-A2-WT1_126-134_ cell line. (**A**) The BD quantibrite beads were used to obtain a standard curve. (**B**) WT1 RMF antigen peptide presentation evaluation of K562-A2-WT1_126-134_ cell.

**
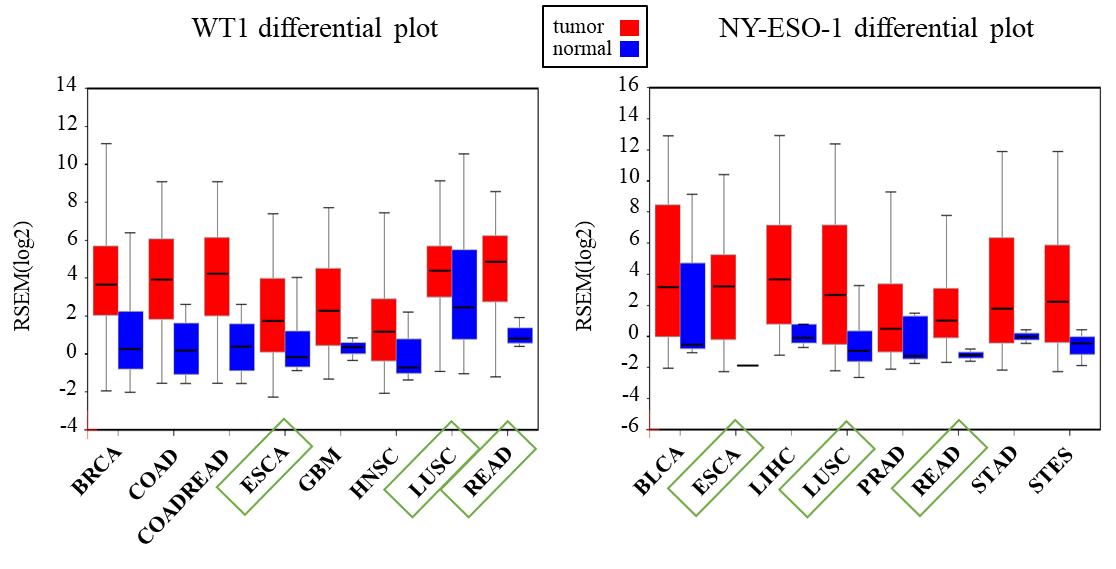

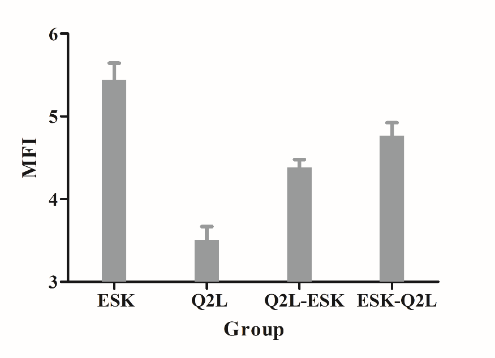
Figure S3.** ESK and Q2L competition-binding on K562-A2-WT1_126-134_ cells determined by flow cytometry.

**Figure S4.** WT1 and NY-ESO-1 expression in different tumors. This figure was acquired from firehose (http://firebrowse.org/). We searched the genes and selected some tumor types to show on the plot in which the fold change of gene expression between tumor and normal data is above three. All the raw data in the website is derived from TCGA database. The Y-axis value represents the expression level according to the RSEM software which can quantify the gene expression. The X-axis shows different tumor types. The red box represents tumor sample and the blue box represents normal sample. The green frames highlight the tumor types in which both of the WT1 and NY-ESO-1 are highly expressed. BRCA: Breast invasive carcinoma; COAD: Colon adenocarcinoma; COADREAD: Colorectal adenocarcinoma; ESCA: Esophageal carcinoma; GBM: Glioblastoma multiforme; HNSC: Head and neck squamous cell carcinoma; LUSC: Lung squamous cell carcinoma; READ: Rectum adenocarcinoma; BLCA: Bladder urothelial carcinoma; LIHC: Liver hepatocellular carcinoma; PRAD: Prostate adenocarcinoma; STAD: Stomach adenocarcinoma; STES: Stomach and esophageal carcinoma.

**Table S1**. Strong binding WT1 protein epitope peptides prediction by NetMHC

| **Peptide length** | **HLA** | **Position** | **Peptide Sequence** | **Affinity(nM)** |
| --- | --- | --- | --- | --- |
| 9 | HLA-A*01:01 | 2-10 | GSDVRDLNA | 950.09 |
|  |  | 152-160 | VTFDGTPSY | 462.50 |
|  |  | 270-279 | YESDNHTTPI | 411.91 |
|  | HLA-A*03:01 | 169-177 | AQFPNHSFK | 28.04 |
|  |  | 240-248 | QMNLGATLK | 47.63 |
|  |  | 287-295 | RIHTHGVFR | 114.80 |
|  | HLA-C*04:01 | 126-134 | RMFPNAPYL | 1697.26 |
|  |  | 417-425 | RWPSCQKKF | 1442.35 |
|  | HLA-C*07:01 | 362-370 | RRFSRSDQL | 41.51 |
|  |  | 393-401 | SRSDHLKTH | 116.55 |
|  |  | 332-340 | KRYFKLSHL | 203.58 |
|  |  | 126-134 | RMFPNAPYL | 224.88 |
|  |  | 221-229 | YSSDNLYQM | 254.67 |
|  |  | 144-152 | IRNQGYSTV | 448.69 |
|  |  | 390-398 | RKFSRSDHL | 482.00 |
|  |  | 365-373 | SRSDQLKRH | 641.58 |
|  |  | 152-160 | VTFDGTPSY | 973.69 |
|  | HLA-C*07:02 | 40-48 | FAPPGASAY | 373.03 |
|  |  | 125-133 | ARMFPNAPY | 543.57 |
|  |  | 126-134 | RMFPNAPYL | 168.72 |
|  |  | 192-200 | QYSVPPPVY | 922.95 |
|  |  | 221-229 | YSSDNLYQM | 1059.15 |
|  |  | 286-294 | YRIHTHGVF | 72.09 |
|  |  | 301-309 | RRVPGVAPT | 1026.12 |
|  |  | 319-327 | EKRPFMCAY | 994.25 |
|  |  | 334-342 | YFKLSHLQM | 764.34 |
|  |  | 362-370 | RRFSRSDQL | 252.83 |
|  |  | 375-383 | RRHTGVKPF | 68.41 |
| 10 | HLA-A*0101 | 100-109 | FTGTAGACRY | 47.14 |
|  |  | 218-227 | RTPYSSDNLY | 457.80 |
|  |  | 270-279 | YESDNHTTPI | 411.91 |
|  | HLA-A*0301 | 168-177 | AAQFPNHSFK | 46.97 |
|  |  | 323-332 | FMCAYPGCNK | 47.02 |
|  |  | 335-344 | KLSHLQMHSR | 80.26 |
|  |  | 399-408 | KTHTRTHTGK | 29.41 |
|  |  | 403-412 | RTHTGKTSEK | 78.61 |
|  |  | 435-444 | HNMHQRNMTK | 41.78 |
| 11 | HLA-A*0101 | 99-109 | QFTGTAGACRY | 187.10 |
|  |  | 317-327 | TSEKRPFMCAY | 24.06 |
|  | HLA-A*0301 | 336-346 | KLSHLQMHSRK | 52.61 |
